# Design and Characterization of Protein E-PilA, a Candidate Fusion Antigen for Nontypeable Haemophilus influenzae Vaccine

**DOI:** 10.1128/IAI.00022-19

**Published:** 2019-07-23

**Authors:** Normand Blais, Don Somers, Denis Faubert, Steve Labbé, Cindy Castado, Carine Ysebaert, Louis-Patrick Gagnon, Josée Champagne, Martin Gagné, Denis Martin

**Affiliations:** a GSK, Rixensart, Belgium; b GSK, Stevenage, United Kingdom; c Institut de Recherches Cliniques de Montréal, Montréal, Quebec, Canada; d GSK, Rockville, Maryland, USA; e GSK, Laval, Quebec, Canada; Washington State University

**Keywords:** crystal structure, *Haemophilus influenzae*, PE, PilA, fusion antigen

## Abstract

Nontypeable Haemophilus influenzae (NTHi) is a pathogen known for being a frequent cause of acute otitis media in children and respiratory tract infections in adults with chronic obstructive pulmonary disease. In the present study, a vaccine antigen based on the fusion of two known NTHi adhesive proteins, protein E (PE) and a pilin subunit (PilA), was developed. The quality of the combined antigen was investigated through functional, biophysical, and structural analyses. It was shown that the PE and PilA individual structures are not modified in the PE-PilA fusion and that PE-PilA assembles as a dimer in solution, reflecting PE dimerization. PE-PilA was found to bind vitronectin by enzyme-linked immunosorbent assay, as isolated PE does. Disulfide bridges were conserved and homogeneous, which was determined by peptide mapping and top-down analysis of PE, PilA, and PE-PilA molecules. Finally, the PE-PilA crystal showed a PE entity with a three-dimensional (3D) structure similar to that of the recently published isolated PE, while the structure of the PilA entity was similar to that of a 3D model elaborated from two other type 4 pilin subunits. Taken together, our observations suggest that the two tethered proteins behave independently within the chimeric molecule and display structures similar to those of the respective isolated antigens, which are important characteristics for eliciting optimal antibody-mediated immunity. PE and PilA can thus be further developed as a single fusion protein in a vaccine perspective, in the knowledge that tethering the two antigens does not perceptibly compromise the structural attributes offered by the individual antigens.

## Abstract (precis)

Nontypeable Haemophilus influenzae (NTHi) is a pathogen known for being a frequent cause of acute otitis media in children and respiratory tract infections in adults with chronic obstructive pulmonary disease. In the present study, a vaccine antigen based on the fusion of two known NTHi adhesive proteins, protein E (PE) and a pilin subunit (PilA), was developed. The quality of the combined antigen was investigated through functional, biophysical, and structural analyses.

## INTRODUCTION

Haemophilus influenzae is a Gram-negative human-specific pathogen able to colonize the upper and lower respiratory tracts. The pathogen is protected by a polysaccharide capsule, but nonencapsulated forms of H. influenzae (nontypeable H. influenzae [NTHi]) exist as well. NTHi is responsible for respiratory tract infections and can be associated with invasive disease. Both adults and children can be affected by NTHi. In adults, it is the major cause of exacerbation in chronic obstructive pulmonary disease (COPD) (1). In children, the clinical manifestations are more diverse. They comprise sinusitis, conjunctivitis, and pneumonia, but NTHi is best known for being the predominant pathogen of chronic and recurrent otitis media (OM) (2–5) and can be considered equivalent to the pneumococcus as an etiologic agent of acute OM (6–11).

The high burden of NTHi diseases in children has motivated the development of prophylactic vaccines targeting this pathogen. Different vaccine antigens have been evaluated in preclinical studies, but none of them was fully satisfactory (12). A major contribution in the prevention of NTHi-induced otitis media was to include H. influenzae-derived protein D (PD) as a carrier for Streptococcus pneumoniae serotypes in a vaccine. In humans, the demonstrated efficacy of PD immunization against NTHi-induced episodes of acute OM was 35.3% in the context of this conjugated vaccine (13, 14). These findings opened interesting perspectives for the control of NTHi-induced diseases but also highlighted the need for an additional NTHi vaccine antigen(s) with the hope to improve efficacy further.

To reinforce the PD approach, we aimed to develop a vaccine antigen based on the fusion of two well-established vaccine candidates, protein E (PE) and a pilin subunit (PilA). Combining these two antigens was meant to afford high strain coverage. PE is a ubiquitous NTHi antigen described to be important for the adhesion of the bacterium to epithelial cells (15–17). It is also known to bind vitronectin, which protects the bacterium from complement attack (18–20), and immunization with a fragment of PE was shown to afford protection in a mouse model of pulmonary clearance (16). Finally, the host immune response is also dampened by the binding of PE to plasminogen (21), which ultimately leads to the degradation of the C3 component of the complement system, after plasminogen has been converted to plasmin. PE is a very well conserved protein, showing 96.9 to 100% identity across Haemophilus influenzae strains, including those that are either encapsulated or that do not have a capsule (17). It was found to be expressed in 98.4% of NTHi strains independently of the growth phase (17). A similar percentage of identity among PE amino acid sequences was found in a more recent study on NTHi strains in COPD patients, distinguishing 14 different haplotypes, but in this study, PE was found in only 83.9% of the strains (22). Nevertheless, such prevalence and conservation rates qualify PE as vaccine antigen for its potential high strain coverage. PilA is another ubiquitous NTHi antigen described to be a conserved protein among H. influenzae isolates (23). It is the major subunit of type IV pili (Tfp) (24), which are hair-like filaments involved in adherence through ICAM-1 (25). Tfp are also involved in twitching motility and biofilm formation (26–28). In this regard, it has recently been shown that anti-PilA antibodies are able to disrupt NTHi biofilms (29). In a recent study, PilA amino acid sequence identity was found to be 89.8% among COPD NTHi isolates, defining 26 different haplotypes (22). All isolates expressed the antigen. In a pilot study, we analyzed 47 COPD strains (from the United States, the United Kingdom, and Spain) and found the PilA occurrence to be 91.3%. The amino acid sequence identity was 76.4% among these strains (unpublished observations). Eventually, in an earlier study by others, PilA was found to be very well conserved among 11 strains, with only two main variability regions being found (26). Antigen variability would normally preclude its use as a vaccine target. However, these variable regions seem to be located outside the main epitope of PilA, which is situated at the C-terminal domain (23). These observations confirmed our choice of using PilA as part of the fusion antigen.

In this study, the quality of the combined PE-PilA fusion antigen was thoroughly investigated, as it is crucial in a vaccine perspective that the two parts of the fusion preserve the integrity of the main epitopes of the individual entities in order to induce the relevant antibody responses. Our results demonstrate that the functional, biophysical, and structural quality attributes of the individual antigens were preserved in the chimeric molecule.

## RESULTS

### The PE-PilA fusion is expressed as a soluble secreted protein.

**FIG 1:**
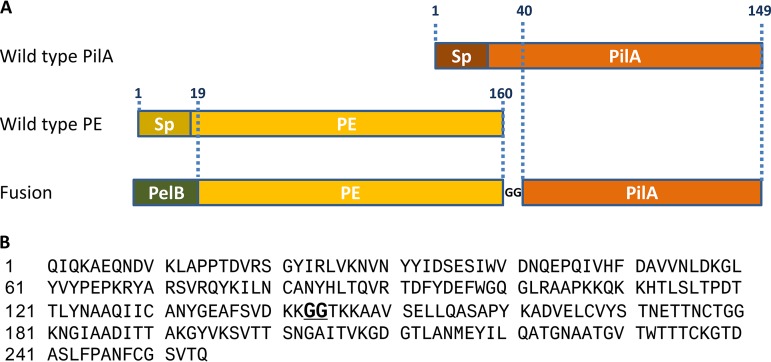
(A) Schematic representation of the fusion between PE and PilA. The full-length wild-type PE and PilA molecules are represented on the top. The wild-type signal peptides (Sp) are highlighted in darker colors, and both were deleted in the final construct. The junction between the two fusion partners is fulfilled by two glycine residues. The PelB signal peptide sequence was inserted at the N terminus of the PE-PilA sequence. The numbers refer to the amino acid numbers in the original PE and PilA sequences. (B) Full amino acid sequence of PE-PilA. The GG hinge between PE and PilA is shown in bold and underlined.

The construct produced in this study was a fusion protein composed of a large part of the PE sequence, a hinge of two glycines, and a large part of the PilA sequence. To direct the protein construct to the bacterial periplasmic space during expression in Escherichia coli, the PelB signal peptide was added at the PE gene N terminus (Fig. 1).

**FIG 2:**
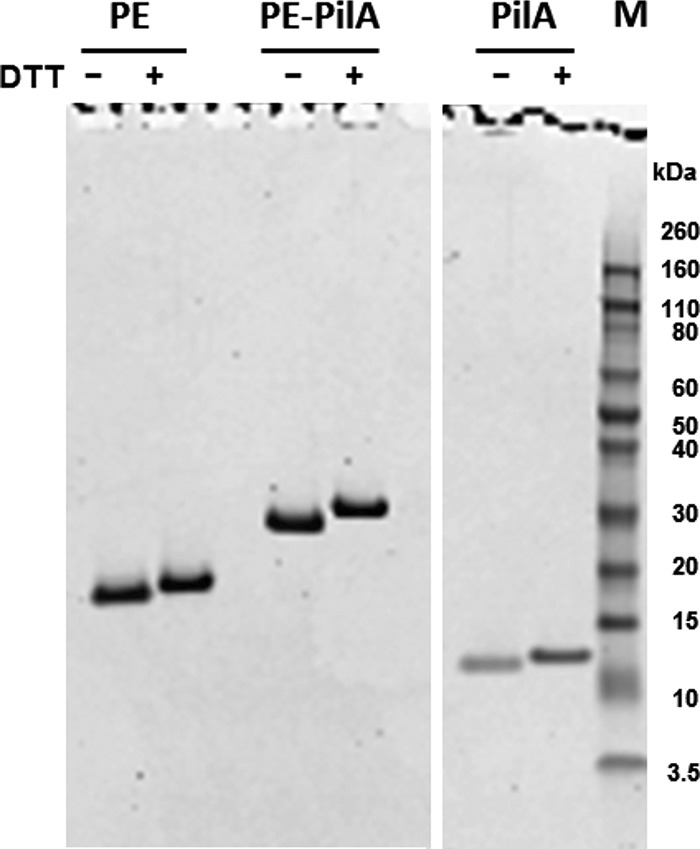
SDS-PAGE analysis of purified PilA, PE, and PE-PilA under nonreducing (without DTT) and reducing (with DTT) conditions. Lane M, molecular weight markers. Their masses (in kilodaltons) are indicated on the right.

After purification, PE, PilA, and PE-PilA were analyzed by sodium dodecyl sulfate-polyacrylamide gel electrophoresis (SDS-PAGE) under nonreducing and reducing conditions (Fig. 2). The band patterns indicated that all three proteins are present as single polypeptides. The absence of multimeric species under nonreducing conditions confirmed that the six cysteine residues of PE-PilA are not implicated in unexpected intermolecular disulfide bridges. Moreover, the observed protein band shift to a higher molecular weight after treatment with a reducing agent suggests the reduction of native intramolecular disulfide bridges.

The correct cleavage of the PelB signal peptide was confirmed by mass spectrometry analyses (see Table S2 in the supplemental material), confirming that the purified PE-PilA was fully processed during expression and successfully translocated into the periplasm.

### PE-PilA is a dimer in solution.

**FIG 3:**
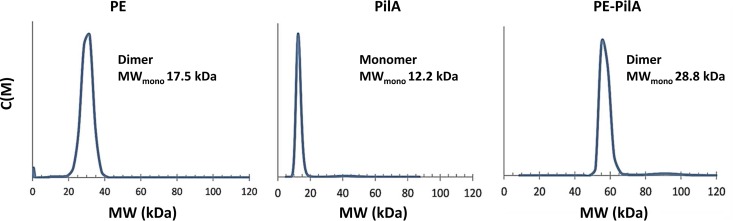
Analytical ultracentrifugation for the determination of the PE, PilA, and PE-PilA size in solution. The molecular weight (MW) of the monomer (MW_mono_) is indicated as an inset in each profile. Consequently, it is also indicated whether the molecule is in monomeric or dimeric form in solution. c(M) is a coefficient that reflects the molecular weight distribution.

Analytical ultracentrifugation analysis of the three purified proteins showed that PilA is monomeric in solution, whereas PE-PilA is present in solution as a dimer, similar to PE (Fig. 3). This suggests that the dimerization properties of PE are not impacted by the presence of PilA as a fusion partner and that PE and PilA are not more prone to form high-molecular-weight oligomers or aggregates when they are expressed in our fusion construct than when they are expressed individually.

### PE-PilA binds vitronectin.

**FIG 4:**
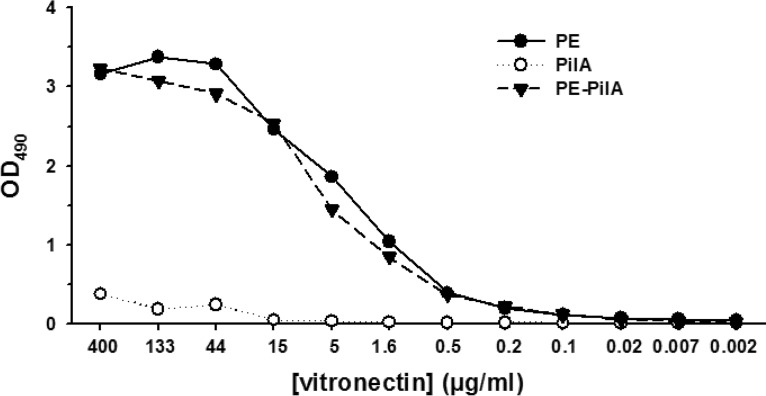
Binding of PE and PE-PilA to vitronectin. Microtiter plates were coated with PE, PilA, or PE-PilA before addition of vitronectin (3-fold serial dilutions starting from 400 μg/ml). Bound vitronectin was detected by peroxidase-labeled sheep antivitronectin antibody followed by *ortho*-phenylenediamine–H_2_O_2_ substrate. The colorimetric reaction was read by determination of the optical density at 490 nm (OD_490_).

Binding to vitronectin is a key function of PE that helps the bacterium to escape complement attacks. We could see that the binding of PE-PilA to vitronectin was similar to that of PE alone (Fig. 4), which indicates that this function of PE is kept despite the fusion with PilA.

### PE and PilA individual structures are not modified in the PE-PilA fusion.

**FIG 5:**
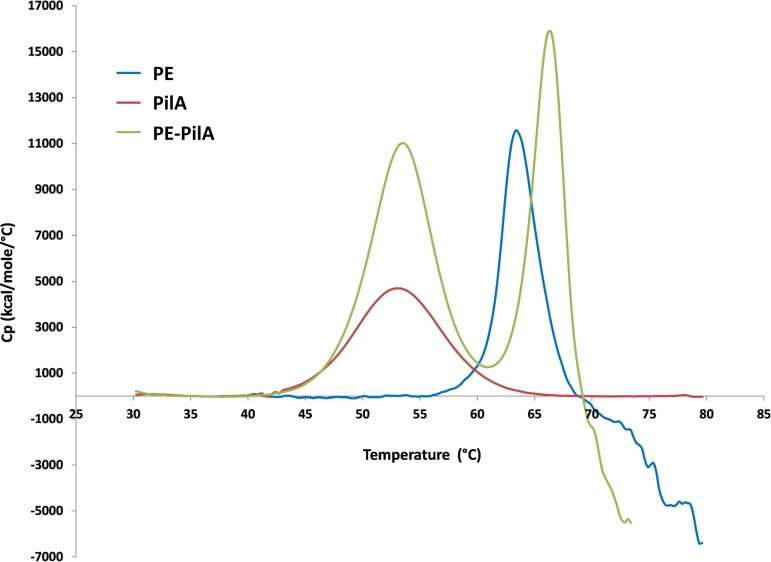
Differential scanning calorimetry thermograms comparing the thermal stability of PE, PilA, and PE-PilA. Cp is the heat capacity.

**(i) Folding of the two fusion entities is conserved in PE-PilA.** The structural stability of folding of the two different entities in the fusion protein was investigated by differential scanning calorimetry (DSC), monitoring the unfolding of the globular structure with temperature (Fig. 5). The melting points of PilA and PE were measured at 53°C and 63°C, respectively. The PE-PilA fusion protein exhibited two distinct melting temperatures at 53.7°C and 66.1°C. These values indicate that PE and PilA unfold at a similar temperature, whether they are separate or in fusion, and that they fold independently without significantly interacting with each other.

**(ii) Disulfide bridges are conserved in PE-PilA.** Disulfide bridges play a key role in the stabilization of the three-dimensional (3D) structure of proteins. The disulfide bridges were analyzed with both bottom-up and top-down proteomic approaches, and two conditions were used: with and without the reduction of disulfide bonds.

The protein sequence coverage of PE obtained with the bottom-up approach after reduction, alkylation, and trypsin was 80% (data not shown). The two cysteines in the PE sequence, i.e., Cys81 and Cys130, could be identified under this condition (they correspond to Cys99 and Cys148 in the full sequence of native PE, respectively). In contrast, after the trypsin digestion only (protein coverage, 48%), the tryptic and semitryptic peptides containing these cysteines were not identified by the protein database-searching algorithm (Mascot). In addition, a peptide containing a disulfide bridge between Cys81 and Cys130 was identified by manual *de novo* sequencing of mass spectrometry/mass spectrometry (MS/MS) spectra (Fig. S1A). The semiquantitative results for both the bottom-up and top-down approaches indicated a relative abundance above 96% for the form with a disulfide bond (data not shown).

The sequence coverage of the PilA sample after reduction, alkylation, and trypsin digestion was 76% (data not shown). Four cysteines, i.e., Cys23, Cys33, Cys92, and Cys105 (corresponding to Cys62, Cys72, Cys131, and Cys144 in the full sequence of native PilA, respectively), were identified. After trypsin digestion only (protein coverage, 49%), these cysteine-containing peptides could not be identified by the protein database-searching algorithm (Mascot). In addition, a peptide containing a disulfide bridge between Cys23 and Cys33 and one between Cys92 and Cys105 was identified by manual *de novo* sequencing of the MS/MS spectra (Fig. S1B and C). The semiquantitative results for both the bottom-up and top-down approaches indicated that the relative abundance of the form containing two disulfide bonds was about 87% (data not shown). Altogether, these data show the existence of two disulfide bridges in the PilA molecule: Cys23-Cys33 and Cys92-Cys105, corresponding to Cys62-Cys72 and Cys131-Cys144 disulfide bridges in complete PilA, respectively.

**FIG 6:**
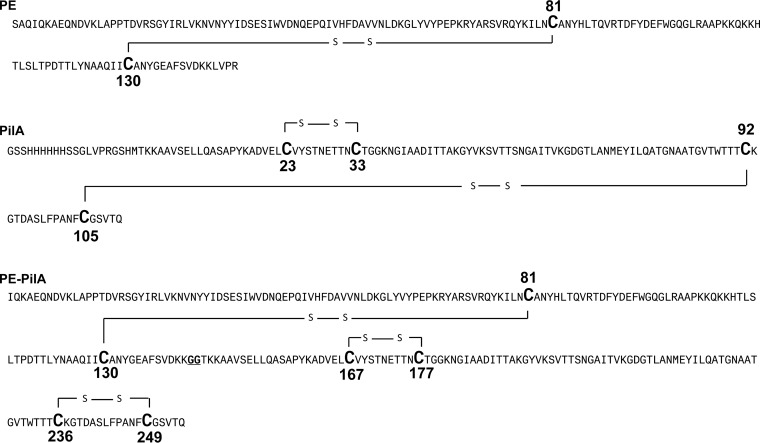
Sequence models of PE, PilA, and PE-PilA showing disulfide bridges. The cysteines involved in disulfide bridges are shown in bold and with a bigger capital letter.

After PE-PilA was subjected to the same trypsin treatments, peptide analysis by mass spectrometry indicated that PE-PilA contains three disulfide bridges that are the same as those identified in individual PE and PilA. In PE-PilA, disulfide bridges exist between Cys81 and Cys130, Cys167 and Cys177, and Cys236 and Cys249 (Fig. 6). The semiquantitative results for both the bottom-up and top-down approaches indicated that the relative abundance of the form containing three disulfide bonds was above 95% (data not shown).

Table S2 shows the identification results of the nonreduced forms of PilA, PE, and PE-PilA by top-down analysis. All proteins were identified using two different fragmentation techniques, and the identification results were highly reliable according to the mass accuracy, the number of identified fragment ions, and the matching scores. These results confirmed the sequence of each protein and the presence of the expected number of disulfide bridges. Table S3 shows the identification results of the reduced protein forms. Again, these results were highly reliable and showed that the reduction of each protein broke the expected disulfide bonds and created free cysteine residues.

**FIG 7:**
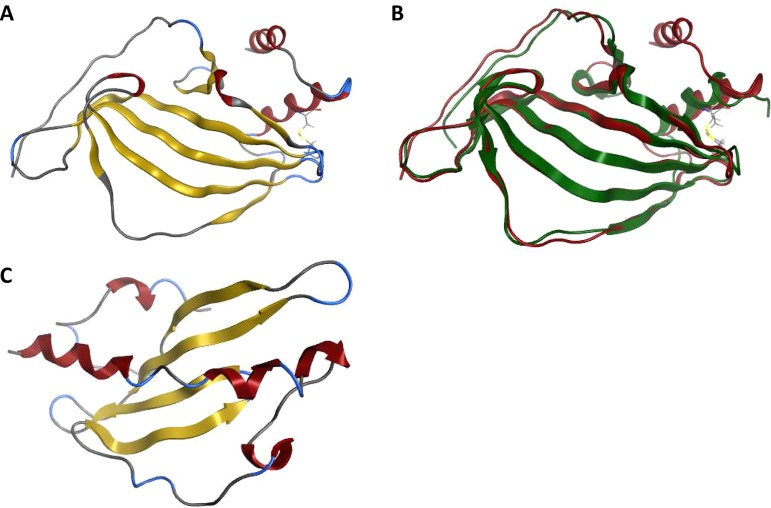
PE structures and PilA model. (A) Crystal structure of isolated His-tagged PE molecule. The β-strands are shown in yellow, and the α-helices are shown in red. The PE monomer comprises a β-sheet with six antiparallel β-strands and two C-terminal α-helices, with one of these being formed by the His tag. A disulfide bond between the main α-helix and the end of β4 is shown. (B) Overlay of isolated His-tagged PE (in red) and the published PE structure (20) (in green) shows a good match between the two structures. (C) Truncated PilA model (based on templates with PDB accession numbers 2HI2 and 1QVE). The β-strands are shown in yellow, and the α-helices are shown in red. PilA consists of a long N-terminal helix lying in the groove of a 4-stranded antiparallel β-sheet.

**(iii) Crystal structures of PE and PE-PilA.** PE crystallized in space group C2 with a monomer in the asymmetric unit, but crystal packing revealed a head-to-tail dimer on the crystallographic 2-fold axis that had an interface area of approximately 950 Å^2^, as determined by the software Protein Interfaces, Surfaces and Assemblies (PISA) (30) run within the COOT model-building tool (31). The PE monomer comprises a β-sheet with six antiparallel β-strands and two C-terminal α-helices, with one of these being formed by heterologous amino acids added in the PE construct used for the crystallization (Fig. 7A). A disulfide bond exists between Cys81 and Cys130, linking the main α-helix with the end of β4, which confirms the mass spectrometry data (see below). The PE crystal structure is comparable to the published structure (20), as revealed by their superposition (Fig. 7B).

PilA crystallization failed to generate any useable crystals for structure determination, so a PilA homology model based on the Neisseria gonorrhoeae (PDB accession number 2HI2) (32) and Pseudomonas aeruginosa (PDB accession number 1QVE) (33) pilins was generated and is shown in Fig. 7C. This model predicts that PilA would comprise a long N-terminal helix lying in the groove of a 4-stranded antiparallel β-sheet.

**FIG 8:**
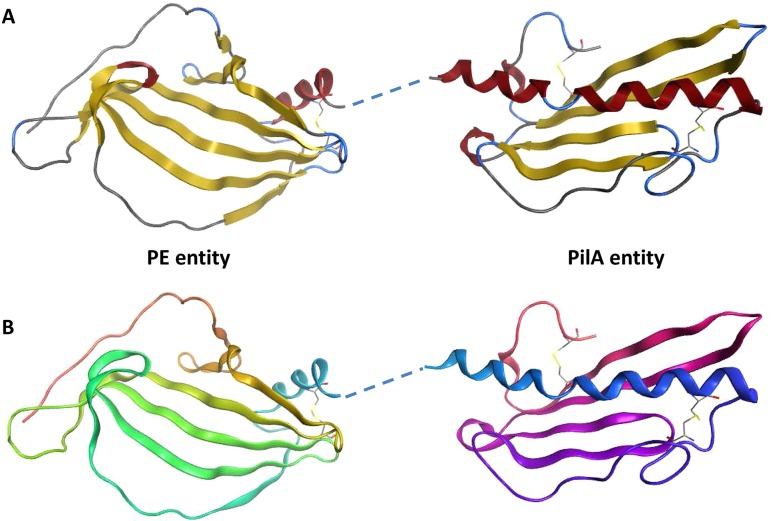
Crystal structure of PE-PilA. (A) The β-strands are shown in yellow, and the α-helices are shown in red. The linker region was not resolved but is shown here as a dashed line linking the two entities. (B) The amino acid succession is shown from orange in the PE entity (N terminus) to pink in the PilA entity (C terminus).

**FIG 9:**
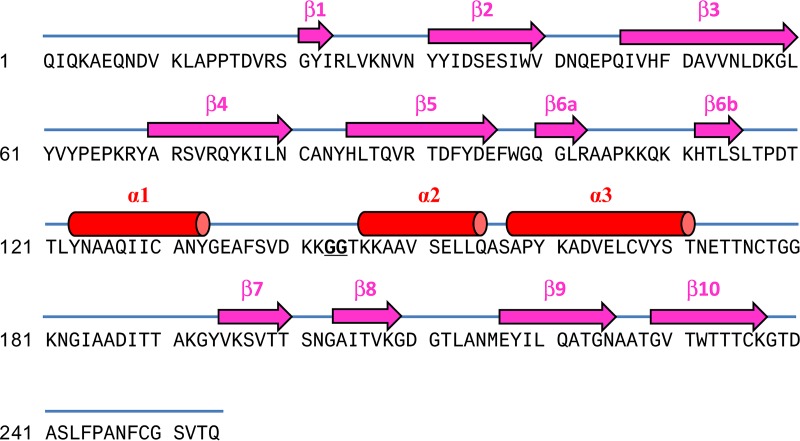
PE-PilA native sequence (signal peptide deleted) and annotation of the secondary structure elements. The β-strands are represented in pink, and the α-helices are represented in red.

The crystal structure of the PE-PilA fusion protein showed that most of the protein was visible, except for three small disordered regions comprising the first seven N-terminal residues (in the PE domain), the linker region (residues 136 to 143), and the C-terminal His tag. PE-PilA also crystallized in space group C2 and had two molecules in the asymmetric unit with a noncrystallographic symmetry dimer interface (area, ∼450 Å^2^) via the PE domain of the fusion protein C terminus. Interestingly, crystal packing analysis revealed a crystallographic 2-fold PE domain dimer, very similar to the one observed in the isolated PE crystal form, with a comparable dimerization interface area (∼870 Å^2^). It is unclear whether this crystallographic dimer represents the biological dimer, but it probably represents a favored interface in the crystallization process. The crystal structure of the PE-PilA fusion protein is shown in Fig. 8, and the secondary structure is shown in Fig. 9.

**FIG 10:**
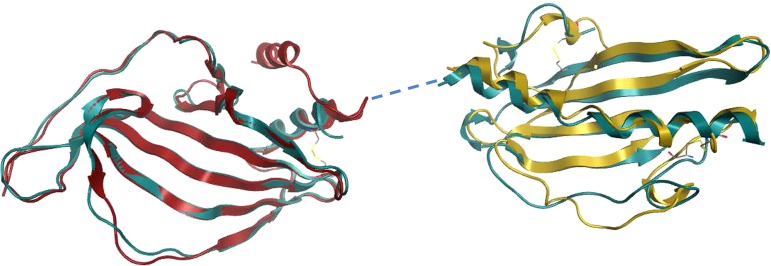
An overlay of the PE-PilA crystal structure (in blue) with the PE structure (in red; root mean square deviation = 1.36), on the one hand, and the 3D PilA model (in gold; root mean square deviation = 2.9), on the other hand, shows a good match between the respective PE and PilA parts of PE-PilA.

The PE and PilA entities of the PE-PilA crystal structure have tertiary structures that closely match the isolated PE crystal structure and PilA homology model, respectively (Fig. 10). In addition, two disulfide bridges in the PilA domain of the fusion protein that corroborate the mass spectrometry data were revealed; one (Cys167-Cys177) links the N-terminal α-helix to the intervening loop of the first β-strand, and the other (Cys236-Cys249) links the final β-strand to the C-terminal loop.

Altogether, our observations support the conclusion that the individual folding of the two entities of the fusion protein is similar to that of the respective individually expressed components.

## DISCUSSION

NTHi vaccine development has proven to be challenging, but our latest success at eliciting partial protection against H. influenzae-induced otitis media with a protein D conjugate vaccine has confirmed its value (13, 14). To further build on this result and reinforce the approach, we have developed a vaccine antigen based on the fusion of two well-established NTHi vaccine candidates, PE and PilA. Combining multiple antigens should increase strain coverage and limit the ability of NTHi to adapt to immune system pressure by presenting antigenic heterogeneity. In addition, the possibility to copurify multiple targets in a single product was found to be critical for vaccine feasibility and accessibility at a reasonable cost. This was even more critical when tackling complex pathological conditions, such as COPD, where targeting multiple pathological targets is inevitable. Passive transfer experiments in chinchillas highlighted the crucial role of antibody responses in the inhibition of NTHi-induced otitis media (34). Therefore, the challenge here was to retain the individual structure and properties of both entities within the fusion structure to ensure that relevant epitopes are maintained to elicit adequate antibody responses.

The resulting 28.8-kDa-molecular-weight fusion protein was a soluble antigen. The fusion of the PelB signal peptide and PE at the N terminus of PilA allowed a high secretion yield in the periplasmic space and the formation of expected disulfide bonds for both antigens. An MS/MS data search for free cysteine residues and potential disulfide bridge scrambling was, encouragingly, unfruitful. Top-down MS/MS analyses confirmed the integrity and homogeneity of the PE-PilA polypeptide sequence. These characteristics represent important quality criteria, since primary structure heterogeneity can impact long-term vaccine stability, and more particularly, an unsuitable modification of relevant B- or T-cell epitopes could undermine the vaccine’s potency. Thermal stability under practical conditions is also a critical vaccine antigen property. In that respect, melting temperatures over 50°C can be considered highly desirable for vaccine application.

Our observations suggest that the two tethered entities behaved independently within the chimeric structure. Particularly, the observation that the first temperature-induced unfolding of the PilA entity was a reversible process and did not cause precipitation of the whole fusion molecule was an indication that the interaction between PE and PilA within the fusion was minimal. However, showing the independent behavior of the two entities was not sufficient. To preserve antigenic properties between recombinant and native molecules, it was important that the conformational integrity of the two antigens be maintained within the chimeric structure. More particularly, the importance of the tertiary structure of PilA was highlighted when it was shown that short PilA peptides were not recognized by antibodies of convalescent-phase sera, whereas longer sections of PilA were (23). The conclusion was that the immunodominant domains of PilA are likely to be discontinuous, emphasizing the need to have a correctly folded PilA for vaccine purposes. In the same work by Novotny et al., the C-terminal part of PilA was defined as the major epitope, and it was determined that the tethering of PilA at the C terminus of its companion entity within a fusion construct was better than that of PilA at the N terminus (23). In line with these observations, PilA was tethered at the C terminus of PE in our construct. It is unclear whether tethered PilA has the same 3D structure as isolated PilA, as the structure of the isolated protein has yet to be determined. Nevertheless, the PilA domain in the chimeric structure reassuringly shared important structural similarities with a model that we elaborated from other type 4 pilins (35). Only the N-terminal hydrophobic α-helix tail was missing in our tethered PilA, but this tail provides both an anchor for the globular head and a hydrophobic surface for the assembly of pilin subunits to form a pilus (36) and as such seems less accessible to antibodies than the globular head and, thus, less immunologically relevant. There were no antigenic data on PE native epitopes, but the goal was to ensure that the PE structural integrity within the fusion protein would not compromise NTHi vaccine performance. In our chimeric structure, we could show in different ways that tethered PE was structurally and functionally similar to isolated PE. Particularly, we observed that PE-PilA was able to inhibit vitronectin binding, a typical PE feature. Consequently, antibodies generated by PE-PilA immunization are expected to inhibit *in vivo* PE-vitronectin binding, thereby neutralizing the possibility for the bacterium to escape complement attack.

Isolated PE and the fusion protein PE-PilA were dimeric in solution, while PilA was monomeric, as judged by analytical ultracentrifugation. The recently published crystal structure of PE (20) showed a noncrystallographic symmetry dimer that the authors suggest is the dimer observed in solution, despite the relatively low interface area (∼625 Å^2^). It was the largest interface in their crystal system, but this type of dimer has not been observed in our crystal systems. We observed a more substantial crystallographic symmetry dimer interface (∼870 Å^2^) in PE-PilA via the PE domain and one of ∼890 Å^2^ for the isolated PE (excluding the C-terminal His tag). The contrasting types of PE dimers observed may simply be due to differences in crystallization conditions. Interestingly, our PE dimers were generated at pH 4 to 4.5, close to the side chain pK_a_ of the key interacting residues Asp52 and Glu54, while the published structure was obtained at pH 6.0.

In conclusion, we have designed and expressed a PE-PilA fusion construct in which both entities keep their original conformation and behave independently. Key functional host factor-binding regions and immunogenic regions appear to be partially or fully surface accessible. These results have encouraged us to further develop PE-PilA in the perspective of using it in vaccines against NTHi-induced diseases. The next steps will consist of immunological analyses to assess the potential of PE-PilA to induce protective immune responses in preclinical models.

## MATERIALS AND METHODS

### Design and construction.

A PE expression vector coding for amino acids 22 to 160 and originating from NTHi strain 772 was kindly provided by K. Riesbeck (Faculty of Medicine, Lund University, Malmö, Sweden). It did not include the precursor sequence coding for the signal peptide, and it contained one mutation compared with the NTHi 772 sequence, introducing one amino acid difference at position 24 in the PE coding sequence, glutamic acid (E) instead of lysine (K). The material characterized included slightly different versions of PE. The plasmid provided was used as the initial template material for subcloning, aiming at replacing exogenous amino acids at the N terminus of the native antigen by the amino acids SAQIQ, QIQ, or IQ and introducing a thrombin cleavage site for His-tag removal at the C terminus.

A PilA expression vector coding for amino acids 40 to 149 and originating from NTHi strain 86-028NP was kindly provided by L. Bakaletz (The Abigail Wexner Research Institute at Nationwide Children’s Hospital and The Ohio State University College of Medicine, Columbus, OH, USA). Its signal peptide as well as a portion of the predicted hydrophobic α-helix in the N terminus was replaced by a 6× His tag, followed by a thrombin cleavage site. This construction was used for the cytoplasmic production of a stand-alone PilA control protein.

The DNA construct used to generate the fusion of PE and PilA was composed of the PelB signal peptide (37), the PE sequence (amino acid 19 to amino acid 160), a hinge of two glycines, and the PilA sequence (amino acid 40 to amino acid 149), as shown in Fig. 1. To ease purification, a 6× His tag was added at the C terminus. A PCR was performed to amplify the PE gene (amino acids 19 to 160) using the PE expression vector as a template and primers CAN544 and CAN546 (Table 1). The DNA sequence corresponding to the PelB signal peptide was incorporated into the 5′ primer (CAN544). To link the *pilA* sequence to the PE gene sequence, the DNA sequence corresponding to two glycines (GG) was added and the N-terminal PilA amino acids were incorporated into the 3′ primer (CAN546). Another PCR was performed to amplify the *pilA* gene (amino acids 40 to 149) using the PilA expression vector as a template with primers CAN545 and CAN535 (Table 1). The DNA sequence corresponding to the C-terminal PE amino acids and GG amino acids was incorporated into the 5′ primer (CAN545) to link the PilA sequence to the PE sequence. The DNA sequence corresponding to the linker GG amino acids and 6× His-tag amino acids was incorporated into the 3′ primer (CAN535). Finally, to generate LVL291, a third PCR was performed to amplify the construct consisting of the PE and *pilA* genes in fusion, with the PelB signal peptide being at the N terminus, a GG linker being between the PE and PilA sequences, and a GG linker being between PilA and the 6× His tag at the C terminus. To achieve this amplification, the products of the two PCRs described above were used as a template with primers CAN544 and CAN535. The DNA sequence corresponding to an NdeI restriction site was incorporated into the 5′ primer, and the DNA sequence corresponding to an HindIII restriction site was incorporated into the 3′ primer. The generated PCR product was then inserted into the pET-26b(+) cloning vector (Novagen) between the NdeI and HindIII sites, giving rise to the LVL291 expression vector. The sequence of the construct was confirmed by DNA sequencing.

**TABLE 1:** PCR primer sequences used for PE, PilA, and PE-PilA amplifications

Primer identifier	DNA sequence (5′-3′)
CAN535	TGTGTGAAGCTTTTAGTGGTGGTGGTGGTGGTGGCCGCCTTGTGTGACACTTCCGCAAAAATTTGC
CAN544	CACACACATATGAAATACCTGCTGCCGACCGCTGCTGCTGGTCTGCTGCTCCTCGCTGCCCAGCCGGCGATGGCCCAGATTCAGAAGGCTGAACAAAATGATGT
CAN545	GCATTTTCAGTTGATAAAAAAGGCGGCACTAAAAAAGCAGCGGTATCTG
CAN546	CAGATACCGCTGCTTTTTTAGTGCCGCCTTTTTTATCAACTGAAAATGC
CAN678	GGAAGTGTCACACAATAAGGCGGCCACCACCACC
CAN679	GGTGGTGGTGGCCGCCTTATTGTGTGACACTTCC

Alternatively, a non-His-tagged version of the construct was made by incorporating a stop codon immediately after the *pilA* gene by site-directed mutagenesis, using LVL291 as a template with primers CAN678 and CAN679 and a QuikChange II site-directed mutagenesis kit (Agilent Technologies, Stratagene Division).

### Expression and purification of recombinant proteins.

**(i) His-tagged PE-PilA, PE, and PilA.** The Escherichia coli BLR(DE3) strain was used for the expression of the PE and PE-PilA constructions, whereas PilA was expressed in the Origami B(DE3) strain. All three His-tagged recombinant proteins were expressed and purified following the same process. Escherichia coli carrying a recombinant plasmid was grown at 37°C in LB broth (Becton, Dickinson and Company). Expression was subsequently induced by cooling the culture to 22°C, before addition of 1 mM IPTG (isopropyl-β-d-thiogalactopyranoside) and overnight incubation. For the PE and PE-PilA constructions, the proteins secreted in the periplasm were purified from a total cell lysate. PilA expressed in the cytoplasm was also recovered from the total cell lysate.

Bacterial cells were resuspended in loading buffer (20 mM HEPES buffer, pH 8.0, 500 mM NaCl, 10 mM imidazole) and disrupted using a Constant system (version 1.1) following the manufacturer’s instructions. His-tagged proteins were purified under native condition by loading the soluble fraction of the cell lysate on an immobilized metal affinity chromatography (IMAC) resin (Bio-Rad Laboratories) that had been preequilibrated in loading buffer. After washing in loading buffer containing 20 mM imidazole, the proteins of interest were eluted by increasing the imidazole concentration to 250 mM. Fractions containing the protein of interest, as determined by SDS-PAGE, were pooled and transferred to a size exclusion chromatography column packed with Superdex 200 (GE Healthcare) in phosphate-buffered saline (PBS; 137 mM NaCl, 2.7 mM KCl, 8.1 mM Na_2_HPO_4_, 1.47 mM KH_2_PO_4_, pH 7.4). Pooled fractions containing the protein of interest were concentrated using a Centricon-10 centrifugal filter (molecular weight cutoff, 10,000; Millipore). Cleavage of the His tag from the purified PilA and PE was performed with a thrombin cleavage capture kit (Novagen) following the manufacturer’s instructions. Following the cleavage and removal of thrombin with the streptavidin agarose supplied in the kit, the mixture was loaded on IMAC affinity resin (Bio-Rad Laboratories) to remove the cleaved His tag and uncleaved PilA. The correctly processed PilA and PE recovered in the flowthrough were concentrated using a Centricon-10 centrifugal filter (molecular weight cutoff, 10,000; Millipore), dialyzed in PBS, and stored at −70°C.

**(ii) Non-His-tagged PE-PilA.** For the purification of non-His-tagged PE-PilA, the protein was produced at a high cell density. Protein extraction by osmotic shock was performed by resuspending the frozen harvested E. coli cell paste in a hypertonic buffer consisting of 24 mM Tris-HCl, 16% (wt/vol) sucrose, 9.9% (wt/vol) glucose, 10 mM EDTA, pH 8. The suspension was centrifuged at 15,900 × *g* for 30 min at room temperature, and the resulting pellet was resuspended in a hypotonic solution consisting of 38 mM MgCl_2_. The mixture was centrifuged at 15,900 × *g* for 30 min at room temperature, and the antigen was recovered in the supernatant. The resulting periplasmic extract was loaded on a strong cationic exchanger resin (SP Sepharose FF; GE Healthcare) that had been equilibrated with 20 mM NaH_2_PO_4_-Na_2_HPO_4_ buffer, pH 7.0. After washing the column with 20 mM NaH_2_PO_4_-Na_2_HPO_4_ buffer, pH 7.0, the antigen was eluted by increasing the concentration of NaCl to 100 mM in the same washing buffer. The antigen present in the SP Sepharose FF eluate was adjusted to 20 mM Tris, pH 8.5, and passed through a strong anionic exchanger resin (Q Sepharose FF; GE Healthcare) that had been equilibrated with 20 mM Tris buffer, pH 8.5. The antigen was recovered in the flowthrough fraction. The Q Sepharose FF flowthrough containing the antigen was transferred to 10 mM KH_2_PO_4_-K_2_HPO_4_ buffer with 0.04% polysorbate 80, pH 6.5.

### SDS-PAGE.

Proteins were analyzed by sodium dodecyl sulfate-polyacrylamide gel electrophoresis (SDS-PAGE) using a Novex bis-Tris 4 to 12% gel (Invitrogen). Samples were prepared following the manufacturer’s instructions with or without dithiothreitol (DTT) to compare reducing and nonreducing conditions. In order to prevent the reduction of disulfide bridges in the nonreduced samples during the separation, no antioxidant was added to the migration tank. The gels were stained in a Coomassie blue-based staining solution.

### Sedimentation velocity by analytical ultracentrifugation.

Protein samples were centrifuged at 25,000 rpm in an AN-60Ti rotor at 15°C in a Beckman-Coulter ProteomeLab XL-1 analytical ultracentrifuge equipped with an absorbance optical detection system. The absorbance profiles at 280 nm were recorded every 5 min. Data were analyzed with Sedfit (version 14.1) software (NIH, Bethesda, MD) using a continuous-size-distribution [*c*(*S*)] model (38).

### DSC.

PE and PilA were first dialyzed overnight in 10 mM K_2_HPO_4_-KH_2_PO_4_, pH 6.5, with 0.04% Tween 80 (1:250 sample-buffer ratio, by volume). After dialysis, the protein concentration was measured by a bicinchoninic assay and adjusted to 300 μg/ml (PE) and 500 μg/ml (PilA). The fusion protein concentration was 740 μg/ml. Analysis was performed on a VP-DSC calorimeter (MicroCal LLC). The samples were scanned from 25°C to 90°C at a 90°C/h scan rate, with 15 min of equilibration being used before each scan. Differential scanning calorimetry (DSC) data were analyzed using Origin (version 7.0) software (OriginLab Corporation). The buffer was used as a reference, and the instrumental baseline was determined with buffer filling both cells prior to subtraction from the raw data scans. Data were normalized for the protein concentration.

### Analysis of disulfide bridges by mass spectrometry.

The presence, position, and relative abundance of disulfide bridges of PE, PilA (His tagged at the N terminus), and PE-PilA were determined by bottom-up and top-down proteomics. The sequences of the three molecules that were used are indicated in Table S2 in the supplemental material. For the bottom-up approach, samples were digested under two different conditions: trypsin digestion after reduction and alkylation or trypsin digestion only. The same conditions were also performed at pH 5.5 to confirm that the disulfide bridges were not formed by disulfide bond scrambling (data not shown). Protein pellets were solubilized in 10 μl of a 6 M urea buffer. For the reduction and alkylation condition, proteins were reduced by adding 2.5 μl of the reduction buffer (45 mM DTT, 100 mM ammonium bicarbonate) for 30 min at 37°C and then alkylated by adding 2.5 μl of alkylation buffer (100 mM iodoacetamide, 100 mM ammonium bicarbonate) for 20 min at 24°C in the dark. Prior to trypsin digestion, 20 μl water was added to reduce the urea concentration to 2 M. A volume of 10 μl of the trypsin solution (5 ng/μl of trypsin [sequencing grade] from Promega, 50 mM ammonium bicarbonate) was added to each sample. Protein digestion was performed at 37°C for 18 h and stopped with 5 μl of 5% formic acid. Protein digests were dried out in a vacuum centrifuge and stored at −20°C until use for liquid chromatography (LC)-MS/MS analysis.

Prior to bottom-up LC-MS/MS analysis, protein digests were resolubilized under agitation for 15 min in 10 to 30 μl of 2% acetonitrile–1% formic acid. The LC column was a C_18_ reversed-phase column packed with a high-pressure packing cell. A 75 μm (inside diameter) Self-Pack PicoFrit fused-silica capillary column (New Objective, Woburn, MA) 15 cm long was packed with the C_18_ Jupiter 5-μm 300-Å reversed-phase material (Phenomenex, Torrance, CA). This column was installed on a NanoLC-2D system (Eksigent) coupled to an LTQ Orbitrap mass spectrometer (Thermo Fisher Scientific, Bremen, Germany) equipped with a Proxeon nanoelectrospray ion source. The buffers used for chromatography were 0.2% formic acid (buffer A) and 100% acetonitrile–0.2% formic acid (buffer B). Peptides were loaded on column at a flow rate of 600 nl/min and eluted with a two-slope gradient at a flow rate equal to 250 nl/min. Solvent B was first increased from 2% to 40% in 11 min and then from 40% to 80% in 3 min. LC-MS/MS data acquisition was accomplished using a four-scan event cycle comprised of a full-scan MS for scan event 1, acquired in the Orbitrap mass spectrometer. The mass resolution for MS was set to 60,000 (at *m/z* 400) and used to trigger the three additional MS/MS events acquired in parallel in the linear ion trap for the top three most intense ions (singly charged ions were excluded). The mass-over-charge ratio range was from 380 to 2,000 for MS scanning with a target value of 500,000 charges and from ∼1/3 of the parent *m/z* ratio to 2,000 for MS/MS scanning with a target value of 20,000 charges. The data-dependent scan events used a maximum ion fill time of 200 ms and 1 microscan. Target ions already selected for MS/MS were dynamically excluded for 6 s. Nanospray and capillary voltages were set to 1.2 to 1.6 kV and 5 to 10 V, respectively. The capillary temperature was set to 225°C. MS/MS conditions were a normalized collision energy of 35 V, an activation *q* of 0.25, and an activation time of 10 ms.

The identification of the tryptic peptides containing the disulfide bridges was performed with manual *de novo* sequencing, whereas the other peptides were identified using Mascot (version 2.1) software (Matrix Science). The mass tolerances for precursor and fragment ions were set to 10 ppm and 0.6 Da, respectively. Trypsin and semitrypsin were used as the enzyme, allowing for up to two missed cleavages. Carbamidomethyl and oxidation of methionine were allowed as variable modifications.

For the top-down approach, the samples were also analyzed under two different conditions: disulfide bridge reduction with DTT treatment and without any treatment. For the reduction condition, the samples were diluted in a 6 M urea buffer and reduced with DTT (45 mM) for 1 h at 45°C. Prior to top-down LC-MS/MS analysis, the samples were diluted in 25% acetonitrile–0.3% trifluoroacetic acid and loaded onto a 50- by 4.6-mm PLRP-S 300 Å column (Agilent Technologies) connected to an Accela pump (Thermo Scientific) and a robotic tool change (RTC) autosampler (Pal Systems). The buffers used for chromatography were 0.1% formic acid (buffer A) and 100% acetonitrile–0.1% formic acid (buffer B). Proteins were eluted with a two-slope gradient at a flow rate of 60 μl/min. Solvent B was first increased from 20% to 60% in 40 min and then from 60% to 70% in 1 min. The high-performance liquid chromatography system was coupled to either an Orbitrap Fusion or a Q Exactive mass spectrometer (Thermo Scientific) through an electrospray ion source. The spray and S-lens voltages were set to 3.6 kV and 60 V, respectively. The capillary temperature was set to 225°C. Full-scan MS survey spectra (*m/z* 900 to 2,000) were acquired in the Orbitrap mass spectrometer with a resolution of 140,000 or 70,000 with a target value of 3e6. The MS/MS fragmentation was performed by both higher-energy collisional dissociation (HCD) and electron transfer/higher-energy collision dissociation (EThcD) with the Orbitrap Fusion mass spectrometer and by HCD only with the Q Exactive mass spectrometer.

The identification of the proteins for the top-down approach was performed with ProSightPC (version 4.0.2.1) software (Thermo Scientific) or ProSight Lite (version 1.4) software (Northwestern University). The mass tolerances for precursor and fragment ions were set to 10 ppm. The only allowed modification was a disulfide bridge (hydrogen loss on cysteine residues).

### Binding to vitronectin.

Microtiter plates (Polysorp; Nunc, Thermo Fisher Scientific) were coated with PE, PilA, or purified PE-PilA fusion protein (10 μg/ml) for 2 h at 37°C. The plates were washed four times with 150 mM NaCl–0.05% polysorbate 20 and blocked for 1 h with PBS–1% bovine serum albumin with agitation. After four washings, serially 3-fold diluted vitronectin (vitronectin from human plasma; Sigma-Aldrich) was added (starting from 400 μg/ml), and the plates were incubated for 1 h at room temperature with agitation. The plates were washed 4 times with 150 mM NaCl–0.05% polysorbate 20. After the washings, bound vitronectin was detected using peroxidase-labeled sheep anti-human vitronectin (US Biological), followed by the addition of *ortho*-phenylenediamine–H_2_O_2_ substrate in 0.1 M citrate buffer, pH 4.5. The reaction was stopped by addition of 1 M HCl, and the plates were read in a microplate reader at 490 nm.

### Crystal structure determination.

PE (at 14.1 mg/ml) in storage buffer A (20 mM HEPES, pH 8.5, 200 mM l-arginine, 200 mM NaCl) was crystallized using hanging-drop vapor diffusion at 20°C by combining the protein with well solution (18.2% polyethylene glycol [PEG] 3350, 1.2 M ammonium sulfate, 0.1 M sodium acetate, pH 4.5) at a 2:1 (protein-well) drop ratio. Crystals were cryoprotected by soaking in cryobuffer (18% PEG 3350, 1.4 M ammonium sulfate, 0.1 M sodium acetate, pH 4.5, 10% glycerol) for approximately 1 h. An initial native diffraction data set (Table S4A, dataset 1) was collected in-house at 100 K using a Rigaku FR-E+ SuperBright generator/Saturn A200 charge-coupled-device detector/Actor robotic system. To facilitate crystallographic phasing, multiple isomorphous replacement was carried out on selected native crystals by soaking them in cryobuffer supplemented with either 1 M KI or 1 M NaBr for 1 h prior to cryocooling and data collection. All diffraction data were processed using the MOSFLM program (39) and scaled using the SCALA program (40) within the CCP4 programming suite (41). Phasing was carried out using the program AutoSHARP by Global Phasing Limited (42), which found and refined heavy-atom sites from the NaBr and KI data sets. Rounds of solvent flattening, ARP/wARP (43) autobuilding, and REFMAC (44) refinement within AutoSHARP resulted in a substantial amount of the single protein in the asymmetric unit being built.

A higher-resolution PE native data set (Table S4A, dataset 2) was subsequently collected at the Diamond synchrotron (station I04-1) at 100 K using a Pilatus 2M detector and processed by the XIA2 expert system (45) (3D-RUN running XDS [46]). Dataset 2 (Table S4A) was used to complete the PE structure determination following final rounds of model building (with COOT [31]) and refinement using the REFMAC program via the CCP4 program suite. Statistics for all the PE data sets collected and the final refined coordinates are given in Table S4A.

PE-PilA (at 13.5 mg/ml) in storage buffer A was crystallized by hanging-drop vapor diffusion at 20°C by combining the protein with well solution (20% PEG 4000, 0.2 M ammonium sulfate, 0.1 M sodium acetate, pH 4.0) at a 1:1 drop ratio. Crystals were cryoprotected by briefly soaking in well buffer with 10% glycerol. A diffraction data set was acquired at 100 K at the ESRF (station ID29) using a Pilatus 6M detector and processed by the synchrotron autoprocessing suite (XDS_PARALLELPROC) (47). The structure was solved by molecular replacement using the previously determined in-house PE crystal structure as a search model in the PHASER program (48), which located both PE subunits of the two chimeric molecules in the asymmetric unit. Autobuilding using ARP/wARP within the CCP4i program suite (49) was then used to build the two PilA subunits. Final rounds of model building (with COOT) and refinement with REFMAC were carried out for structure completion. Statistics for the data set collected and refined coordinates are given in Table S4B.

### PilA model building.

Optimal structural templates were identified using analysis with the BLAST program (50) against the sequences in the Protein Data Bank (PDB; www.rcsb.org) (51). Two structural templates were identified for NTHi PilA protein homology modeling: type IV pilin from Neisseria gonorrhoeae (PDB accession number 2HI2) (32) and pilin from Pseudomonas aeruginosa (PDB accession number 1QVE) (33). Sequence-to-structure prediction was performed with the NTHi PilA protein sequence and the identified structural templates for analysis in Molecular Operating Environment (MOE; version 2016.0804; Chemical Computing Group, Inc., Canada). The sequence alignment provided by MOE was manually edited by aligning the predicted secondary structures (for PilA, the prediction was performed using the PsiPred program [52]) and known secondary structures (for the structural templates). The modeling analysis was performed using the optimized sequence alignment and the homology modeling tool of MOE. The Amber10:EHT force field was selected, a structural template with PDB accession number 2HI2 was used as the main structural template, and the structural template with PDB accession number 1QVE was used for the C-terminal modeling of the NTHi PilA sequence (option “fine” for intermediates and final model refinement with a root mean square gradient of 0.1). After 3D structure prediction, the MOE energy minimization tool (default parameters) was applied to optimize the PilA model.

### Accession number(s).

The PE crystal structure was deposited in the Protein Data Bank under accession number 6GUS. The PE-PilA crystal structure was deposited in the Protein Data Bank under accession number 6GUT.

## Supplementary Material

Supplemental file 1
