# Supplementary material for: Design and Characterization of Protein E-PilA, a Candidate Fusion Antigen for Nontypeable Haemophilus influenzae Vaccine
Source: Infect Immun. 2019 Jul 23;87(8):e00022-19. doi: 10.1128/IAI.00022-19 (PMC6652776; doi:10.1128/IAI.00022-19)
Supplement: Supplemental file 1 [file IAI.00022-19-s0001.pdf]

Supplementary Figure 1. A. Annotated HCD MS/MS spectrum of the PE peptide containing the disulfide bond between Cys81 and Cys130. The precursor m/z value is 1200.34597 and mass accuracy with the annotated peptide is 0.5 ppm. B. Annotated EThcD MS/MS spectrum of the PilA peptide containing the disulfide bond between Cys23 and Cys33. The precursor m/z value is 701.63846 and mass accuracy with the annotated peptide is 0.1 ppm. C. Annotated HCD MS/MS spectrum of the PilA peptide containing the disulfide bond between Cys92 and Cys105. The precursor m/z value is 1175.7976 and mass accuracy with the annotated peptide is 1.6 ppm.

A

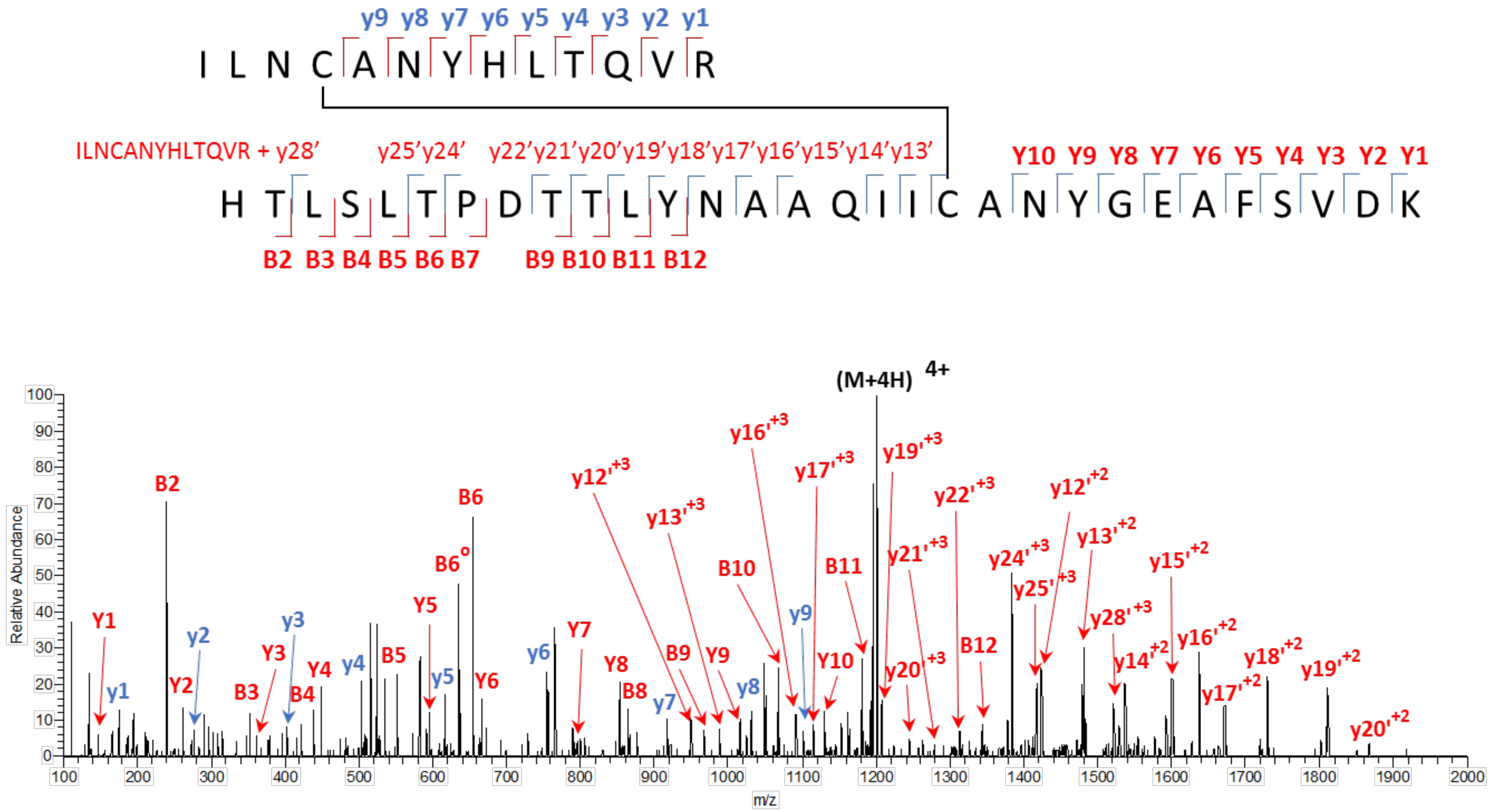

B

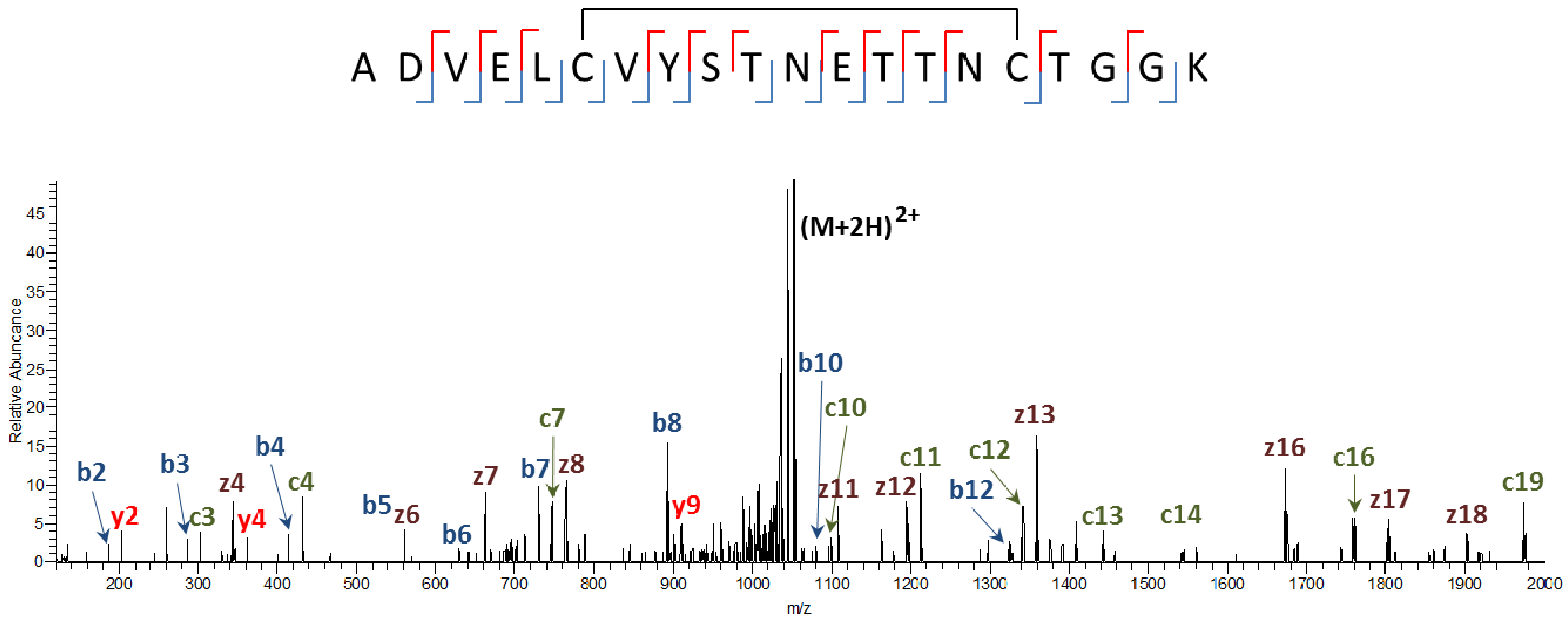

A D V E L C V Y S T N E T T N C T G G K
(M+2H)2+
b2
y2
b3
c3
z4
y4
b4
c4
b5
z6
b6
z7
b7
c7
z8
b8
y9
b10
c10
z11
z12
c11
b12
c12
z13
c13
c14
z16
c16
z17
z18
c19
Relative Abundance
m/z

C

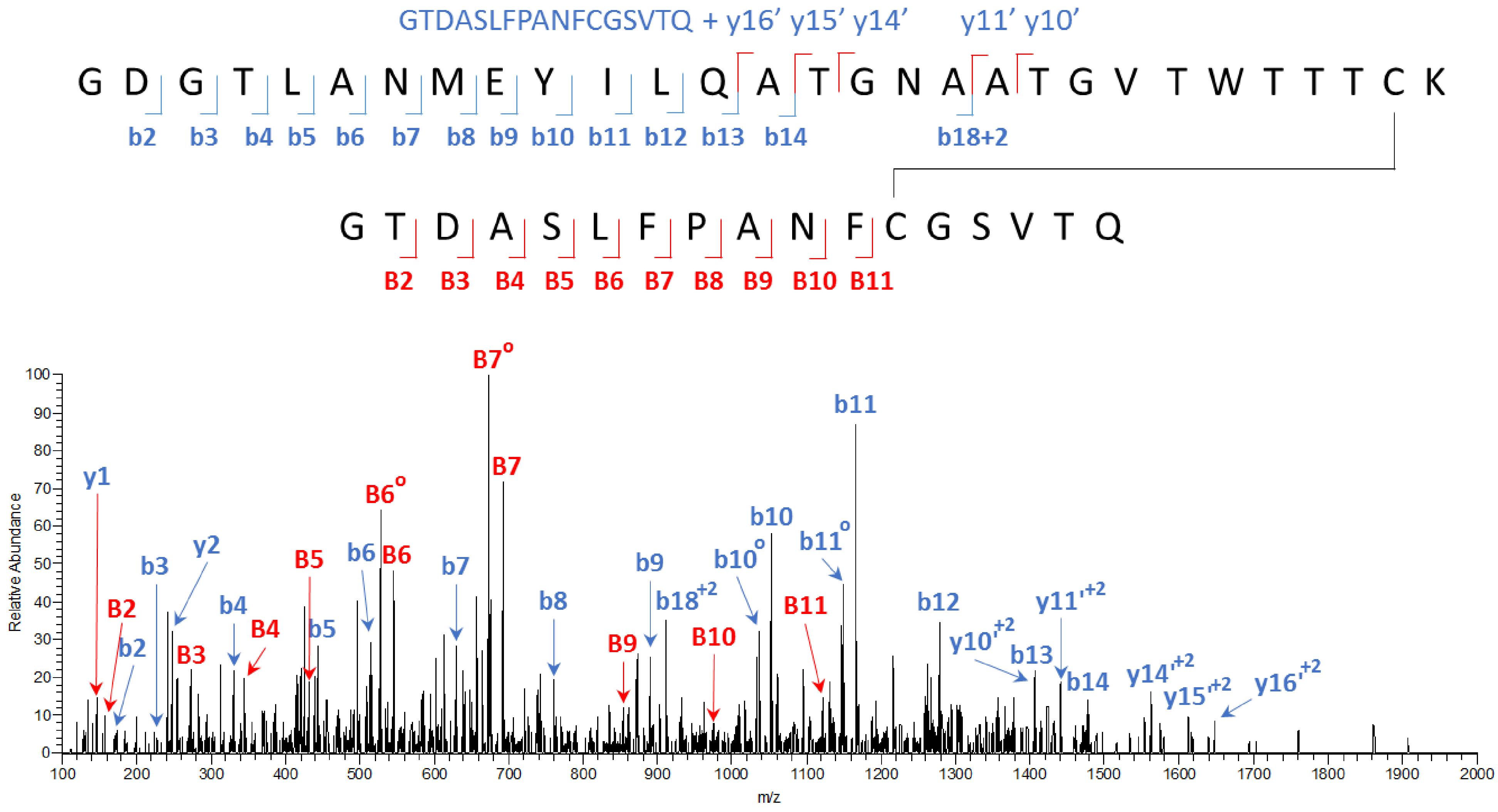
GTDASLFPANFCGSVTQ + y16' y15' y14'
y11' y10'
G D G T L A N M E Y I L Q A T G N A A T G V T W T T T C K
b2 b3 b4 b5 b6 b7 b8 b9 b10 b11 b12 b13 b14 b18+2
G T D A S L F P A N F C G S V T Q
B2 B3 B4 B5 B6 B7 B8 B9 B10 B11
Relative Abundance
m/z
B7°
b11
B7
y1
B6°
b10
b11°
y2
b3
B5
b6
B6
b7
b9
b10°
b18+2
b4
B2
B4
b5
B11
b12
y11'+2
b2
B3
B9
B10
y10'+2
b13
b14
y14'+2
y15'+2
y16'+2

Identified protein	Experiment	Search Type	Sequence	Number of Matching Fragments	PTMs	Theoretical Mass	Observed Mass Da	Mass Diff Da	Mass Diff ppm	B-ions	C-ions	Y-ions	Z-ions	PCS score	P Score	% Fragments Explained	% Residue Cleavages
PilA	HCD fragmentation	ProSight Lite	GSSHHHHHHSSGLVPRGSHMTKKAAVSELLQASAPYKADVELCV<br>YSTNETTNCTGGKNGIAADITTAKGYVKSVTTSNGAITVKGDGTLA<br>NMEYILQATGNAATGVTWTTTCKGTDASLFPANFCGSVTQ	72	disulfide bond (x2)	13374.44	13374.49	0.05	3.7	53		19		943	1.6E-80	16	48
PilA	EThcD fragmentation	ProSight Lite	GSSHHHHHHSSGLVPRGSHMTKKAAVSELLQASAPYKADVELCV<br>YSTNETTNCTGGKNGIAADITTAKGYVKSVTTSNGAITVKGDGTLA<br>NMEYILQATGNAATGVTWTTTCKGTDASLFPANFCGSVTQ	72	disulfide bond (x2)	13374.44	13374.49	0.01	1.0	21	31	2	18	730	3.1E-64	39	34
PE	HCD fragmentation	ProSight Lite	SAQIQKAEQNDVKLAPPTDVRSGYIRLVKNVNYYIDSESIWVDNQ<br>EPQIVHFDAVVNLDKGLYVYPEPKRYARSVRQYKILNCANYHLTQ<br>VRTDFYDEFWGQGLRAAPKKQKKHTLSLTPDTTLYNAAQIICANY<br>GEAFSVDKKLVPR	58	disulfide bond (x1)	17071.78	17071.77	-0.02	-0.9	32		26		414	1.2E-39	6	32
PE	EThcD fragmentation	ProSight Lite	SAQIQKAEQNDVKLAPPTDVRSGYIRLVKNVNYYIDSESIWVDNQ<br>EPQIVHFDAVVNLDKGLYVYPEPKRYARSVRQYKILNCANYHLTQ<br>VRTDFYDEFWGQGLRAAPKKQKKHTLSLTPDTTLYNAAQIICANY<br>GEAFSVDKKLVPR	36	disulfide bond (x1)	17071.78	17071.74	-0.05	-2.6	3	29	4	0	295	3.6E-30	36	24
PE-PilA	EThcD fragmentation	ProSight Lite	IQKAEQNDVKLAPPTDVRSGYIRLVKNVNYYIDSESIWVDNQEPQI<br>VHFDAVVNLDKGLYVYPEPKRYARSVRQYKILNCANYHLTQVRTD<br>FYDEFWGQGLRAAPKKQKKHTLSLTPDTTLYNAAQIICANYGEAFS<br>VDKKGGTKKAAVSELLQASAPYKADVELCVYSTNETTNCTGGKNG<br>IAADITTAKGYVKSVTTSNGAITVKGDGTLANMEYILQATGNAATG<br>VTWTTTCKGTDASLFPANFCGSVTQ	40	disulfide bond (x3)	27628.84	27628.85	0.00	0.4	12	24	3	1	506	7.5E-47	55	12
PE-PilA	HCD fragmentation	ProSight Lite	IQKAEQNDVKLAPPTDVRSGYIRLVKNVNYYIDSESIWVDNQEPQI<br>VHFDAVVNLDKGLYVYPEPKRYARSVRQYKILNCANYHLTQVRTD<br>FYDEFWGQGLRAAPKKQKKHTLSLTPDTTLYNAAQIICANYGEAFS<br>VDKKGGTKKAAVSELLQASAPYKADVELCVYSTNETTNCTGGKNG<br>IAADITTAKGYVKSVTTSNGAITVKGDGTLANMEYILQATGNAATG<br>VTWTTTCKGTDASLFPANFCGSVTQ	24	disulfide bond (x3)	27628.84	27628.80	-0.04	-1.4	22		2		503	1.2E-46	36	10

Table S2. **TOP DOWN RESULTS WITH NO REDUCTION OF DISULFIDE BRIDGES**

Identified protein	Sequence	Number of Matching Fragments	PTMs	Theoretical Mass	Observed Mass Da	Mass Diff Da	Mass Diff ppm	B-ions	Y-ions	PCS score	P Score	% Fragments Explained	% Residue Cleavages
PilA	GSSHHHHHHSSGLVPRGSHMTKKAAVSELLQASAPYKADVELCVYSTNE<br>TTNCTGGKNGIAADITTAKGYVKSVTTSNGAITVKGDGTLANMEYILQAT<br>GNAATGVTWTTTCKGTDASLFPANFCGSVTQ	101	none	13378.47	13378.45	-0.02	-1.5	75	26	1333	3.0E-110	16	64
PE	SAQIQKAEQNDVKLAPPTDVRSGYIRLVKNVNYYIDSESIWVDNQEPQIV<br>HFDAVVNLDKGLYVYPEPKRYARSVRQYKILNCANYHLTQVRTDFYDEF<br>WGQGLRAAPKKQKKHTLSLTPDTTLYNAAQIICANYGEAFSVDKKLVPR	23	none	17073.80	17073.83	0.04	2.1	12	11	353	8.9E-35	27	16
PE-PilA	IQKAEQNDVKLAPPTDVRSGYIRLVKNVNYYIDSESIWVDNQEPQIVHFD<br>AVVNLDKGLYVYPEPKRYARSVRQYKILNCANYHLTQVRTDFYDEFWGQ<br>GLRAAPKKQKKHTLSLTPDTTLYNAAQIICANYGEAFSVDKKGGTKKAAVS<br>ELLQASAPYKADVELCVYSTNETTNCTGGKNGIAADITTAKGYVKSVTTSN<br>GAITVKGDGTLANMEYILQATGNAATGVTWTTTCKGTDASLFPANFCGS<br>VTQ	30	none	27634.89	27634.93	0.05	1.7	23	7	343	5.1E-34	17	12

Table S3 TOP DOWN RESULTS WITH REDUCTION OF DISULFIDE BRIDGES

Table 4A. PE Crystallography data collection and analysis.

	**PE (Dataset 1)**		**PE (Dataset 2)**		**PE (NaBr)**		**PE (KI)**	
**Parameter**	**Overall**	**Outer shell**	**Overall**	**Outer shell**	**Overall**	**Outer shell**	**Overall**	**Outer shell**
Space group	$I4_1$		$I4_1$		$I4_1$		$I4_1$	
a (Å)	77.64		77.67		77.26		77.13	
b (Å)	77.64		77.67		77.26		77.13	
c (Å)	65.73		66.13		66.29		67.58	
α (°)	90		90		90		90	
β (°)	90		90		90		90	
γ (°)	90		90		90		90	
Low resolution limit (Å)	27.45	2.11	30.75	1.97	27.32	2.11	25.42	2.32
High resolution limit (Å)	2.00	2.00	1.92	1.92	2.00	2.00	2.20	2.20
R_{merge}	0.051	0.398	0.029	0.466	0.067	0.820	0.096	0.517
R_{meas} within I+/I-	0.061	0.473	0.034	0.545	0.073	0.883	0.103	0.559
R_{meas} all I+/I-	0.060	0.476	0.034	0.545	0.074	0.877	0.121	0.558
R_{pim} within I+/I-	0.032	0.249	0.018	0.281	0.027	0.325	0.039	0.211
R_{pim} all I+/I-	0.023	0.180	0.013	0.201	0.019	0.231	0.032	0.150
Fractional partial bias	-0.029	-0.107	0	0	-0.022	-0.233	-0.039	-0.181
Total number of reflections	91978	7787	103007	7787	187156	26975	139904	20112
Number of unique	13226	1084	14861	1084	13223	1920	10123	1464

reflections								
Mean (I)/σ(I)	17.7	3.9	28.1	3.3	19.3	2.9	16.3	5.0
Completeness	99.8	100.0	98.6	97.6	99.8	99.9	99.9	100.0
Multiplicity	6.9	7.2	6.9	7.2	14.2	14.0	13.8	13.7
Phasing								
Resolution (Å)					2.00		2.00	
No. of sites					4		4	
Phasing power[b]								
Acentric					1.928		2.130	
Centric					1.423		1.672	
Rcullis								
Acentric					0.544		0.423	
Centric					0.597		0.418	
Refinement statistics								
Rfactor			0.198					
Rfree			0.249					
No atoms			1267					
RMS bond lengths (Å)			0.008					
RMS bond angles (°)			1.291					
Wilson (Truncate style) B-factor ($Å^2$)	35.135		39.0		40.464		39.588	
Average B-Factor ($Å^2$)								

Protein			58.7						
Ligand/Ion			73.2						
Water			61.5						
Ramachandran analysis									
Favoured (%)			95.1						
Allowed (%)			4.2						
Outliers (%)			0.7						
Molprobity ClashScore			3.84						

Each dataset was collected from one crystal.

[a]Highest-resolution shell is shown in parenthesis.

[b]Phasing power calculated as r.m.s. ($|F_h|/E$), where $|F_h|$ is the heavy-atom structure factor amplitude and E the residual lack of closure error.

[c]Acentric reflections. R_{Cullis} calculated as ($\Sigma|E|/\Sigma||F_{PH}| - |F_P||$), where F_{PH} is the amplitude of the protein plus the heavy atom and F_P is the amplitude of the protein.

Table 4B. PE-PilA Crystallography data collection and analysis.

	**PE-PilA**	
**Parameter**	**Overall**	**Outer shell**
Space group	C2	
a (Å)	128.618	
b (Å)	83.458	
c (Å)	59.049	
α (°)	90	
β (°)	102.52	
γ (°)	90	
Low resolution limit (Å)	47.96	1.69
High resolution limit (Å)	1.63	1.63
R_{merge}	0.062	0.543
R_{meas} within I+/I-	0.074	0.649
R_{meas} all I+/I-	0.074	0.649
R_{pim} within I+/I-	0.039	0.351
R_{pim} all I+/I-	0.039	0.351
Fractional partial bias	0	0
Total number of reflections	256262	24401
Number of unique reflections	75506	7386
Mean (I)/σ(I)	10.4	2
Completeness	99.5	99.4
Multiplicity	3.4	3.3
Refinement statistics		
Rfactor	0.179	
Rfree	0.209	
No atoms	4332	
RMS bond lengths (Å)	0.008	
RMS bond angles (°)	1.245	
Wilson B-factor ($Å^2$)	24.57	
Average B-Factor ($Å^2$)		
Protein – Chain A	30.0	
Protein – Chain B	24.9	
Ligand/Ion	51.0	
Water – Chain V	40.4	
Water – Chain W	39.7	
Ramachandran		

analysis		
Favoured (%)	98.5	
Allowed (%)	1.5	
Outliers (%)		
Molprobity ClashScore	3.03	

Each dataset was collected from one crystal.

[a]Highest-resolution shell is shown in parenthesis.

[b]Phasing power calculated as r.m.s. ($|F_h|/E$), where $|F_h|$ is the heavy-atom structure factor amplitude and E the residual lack of closure error.

[c]Acentric reflections. R_{Cullis} calculated as ($\Sigma|E|/\Sigma||F_{PH}| - |F_P||$), where F_{PH} is the amplitude of the protein plus the heavy atom and F_P is the amplitude of the protein.
